# Quality of life among residents of Gaza, Palestine: the predictive role of mental distress, fear of COVID-19, and social support

**DOI:** 10.1186/s40359-024-01642-8

**Published:** 2024-03-15

**Authors:** Suhayla Said Jalala, Guido Veronese, Marwan Diab, Yasser Abu Jamei, Rawya Hamam, Ashraf Kagee

**Affiliations:** 1 Department of Human Sciences and Education “R. Massa”, University of Milano-Bicocca, Milan, Italy; 2 Department of Psychology, Stellenbosch University, Stellenbosch, Matieland South Africa; 3 Gaza Community Mental Health Program, Gaza, Palestine

**Keywords:** Quality of life, Mental distress, Fear of COVID-19, Social support, Political instability, Gaza strip

## Abstract

**Background:**

Living under siege and deteriorated health, social, educational, and economic conditions and isolation with scarce opportunities to fulfil basic needs and aspirations affect the civil population's mental health and perceived quality of life. In this cross-sectional investigation, we explored the consequences of mental distress, fear of COVID-19, and social support for QoL in the Gaza strip.

**Methods:**

Nine hundred seventy nine (32.9% males; 67.1% females; mean age was 35.2 years; s.d. = 11.4) adults were recruited in the Gaza strip. We used the *Fear for COVID-19 scale (FCS-19), The WHOQOL-BREF Scale, Berlin Social Support Scale (BSSS), Depression Anxiety and Stress Scale (DASS).* Pearson correlation coefficient was computed to assess relationships between quality of life, fear of COVID19, mental distress, and social support; a hierarchical regression analysis was used to assess the association between QoL as the dependent variable and demographic variables and fear of COVID19, mental health, and social support as the independent variables.

**Results:**

QoL was positively associated with perceived emotion, instrumental, and support seeking. Depression, anxiety, stress, and fear of COVID19 were negatively associated with quality of life. Gender was significantly associated with lower QoL. The study highlighted that the level of fear of COVID-19 was negatively influencing individuals' quality of life (QoL). This fear was negatively associated to psychological distress, gender, place of residence, and family type. Lower-educated and poorer participants had lower QoL scores. Conversely, female gender was notably linked to a lower QOL. The hierarchical regression confirmed that COVID-19 was an added burden for the Palestinian population. The fear of COVID-19 term added a 6.2% variance in QoL. In the final analysis, all predictors were statistically significant, with the fear of COVID-19 term recording a higher contribution of 22.5%, followed by depression term with 21.5%, perceived emotional 18.5%, income at 15.4%, and perceived instruments at 14.8% towards QoL.

**Conclusions:**

Practitioners and policymakers must consider the severe violation of human rights when developing psychosocial programs to intervene in the COVID-19 crisis.

## Introduction

COVID-19 has swept through every country globally, particularly in crisis zones with limited resources and capacities [1–4]. The Gaza Strip, one of the world's most densely inhabited areas, is located on the Mediterranean Sea's south-eastern side, with 2 million people living in a 365-square-kilometre territory [5]. Being overcrowded with few resources coupled with a 16-year blockade by Israel and going population has exacerbated the deterioration of health, social, educational, and economic conditions, as well as the identity of Gazans [6–8]. As a result, people in Gaza suffer from a high unemployment rate [9, 10], poverty, poor infrastructure [10, 11], and a fragile health system [12]. They also experience suffering from the Israeli-imposed restrictions on access to agricultural land and fishing waters [13].

Armed conflicts have a negative influence on people's social determinants of mental health and well-being [14, 15], and affects their ability to meet their basic needs such as health, economic, social, and educational demands [16–18]. According to several studies, the quality of life in the occupied Palestinian territory (oPt) was found to be among the lowest of any population in the world, including the physical and psychological [19–21], and environmental domain [19].

The healthcare system in Gaza Strip is already overburdened and has deteriorated significantly as a result of repeated rounds of violence with Israel in 2008, 2012, and 2014, 2023 the Great March of Return demonstrations, and the overall situation of unrest [22, 23]. On Friday, May 10, 2021, an Israeli escalation occurred while the oPt was still coping with a surge of coronavirus cases in Gaza, with the number of active cases exceeding 60% in April 2021. During the fighting, follow-up on COVID-19 preventative measures, vaccination and testing have been severely hampered. Moreover, the central testing facility and the single vaccination centre in northern Gaza were damaged [24]. This situation makes Gaza weak and fragile in responding effectively to the global crisis of the COVID-19 pandemic [4].

Since the Coronavirus outbreak, most governments worldwide have taken quick actions to halt the spread of the virus [25–27]. In Gaza, the Palestinian Authority implemented several restrictions, including travel restrictions, and social isolation, as many stayed at home and many employees worked from home [22, 28, 29].

Several studies have addressed the impact of the COVID-19 pandemic, including its effect on the mental health of several groups of people in fragile and conflict-affected settings [30–35]. Following COVID-19 infection, there is evidence of increased levels of indirect effects of COVID-19 on general mental health, such as posttraumatic stress in hospitalized patients or health providers, depression, anxiety [36], acute panic, obsessive behaviours, paranoia, fear, and anger [37, 38] as well as negative symptoms impacting overall mental health. The COVID-19 pandemic has had a global psychosocial impact, creating mass fear of “corona phobia”, economic burdens, and financial losses [32, 39]. As a result, during the pandemic, families in Gaza have encountered several problems that have harmed their well-being, including domestic violence, conflict, and divorce. Studies have shown that the most affected groups were young, female, married, and not well-educated [39, 40].

A study by Vujčić and colleagues (2021) assessed the mental health impact of COVID-19 on the adult population in Serbia during the state of emergency and lockdown. Out of 1057 participants, high rates of depression (28.9%), anxiety (36.9%), and stress (38.1%) were reported. Factors such as uneasiness from COVID-19 news, helplessness, perceived likelihood of death, and COVID-19 symptoms were associated with higher mental health symptoms. Smoking, student status, age, and socioeconomic status also played a role [41].

Another study in Serbia examined mental health among healthcare workers during the COVID-19 pandemic. Results showed that resilience and capacity for mentalizing were linked to depression, anxiety, and stress levels. Higher resilience and hypermentalizing were associated with lower mental health symptoms, while hypomentalizing was linked to higher symptoms. Socioeconomic status also influenced mental health [42, 43].

Social support is essential to an individual's well-being and plays a vital role in crisis management [44–48], either by supporting avoidance strategies or by encouraging a treatment strategy [49].

Established studies demonstrate that social support is a moderating variable in the association between the reaction to stress and psychological distress [50–56]. “Social support is” social interactions or relationships that provide individuals with actual assistance or include individuals in a social system who are believed to provide care, love, or a sense of connection to a social group of value.” [57–59]. Individuals seeking social support increase their social resources by offering empathy or decreasing their feelings of loneliness [60–62].

In contrast, lack of social support and feelings of social isolation are the most important predictors of psychological illnesses such as posttraumatic stress after exposure to crises or traumatic events [63–66]. Reavell and Fazil [42] found that high mental health problems were linked to increased susceptibility to injury, while social support was crucial in reducing symptoms of traumatization and depression. Furthermore, in a study on Palestinian adolescents, Al-Sheikh and Thabet [63] found that overall traumatic events were negatively correlated with social support. Similarly, Labrague and Santos [47] and Pietrzak et al. [60] showed that higher social support is linked with lower anxiety levels related to the COVID-19 pandemic and can buffer against the onset and maintenance of posttraumatic stress disorder (PTSD) symptoms and other mental health problems over time.

According to the previous literature review, we sought to explore the consequences of mental distress and COVID-19-related burdens in a society characterized by ongoing violence and political instability and the role of social support on the deterioration of quality of life (QoL). Our leading hypotheses were that QoL was associated with higher social support (H1), while QoL would deteriorate as a result of mental distress (depression, anxiety, and stress) (H2). Furthermore, fear of COVID-19 would be negatively associated with QoL (H3), mainly in the population with lower socioeconomic status (H4). Finally, we explored gender differences, hypothesizing that women's QoL may have been more affected during the pandemic (H5). Ultimately, our study aimed to explore the predictors of the quality of life in the Gaza strip during the COVID-19 pandemic.

## Methods

This study adhered to the guidelines of the Declaration of Helsinki.

### Study design and population

Approval was granted by the Ethics Committee of Stellenbosch University (Date: 7–12-2020/No: REC-2020–17479) and the Helsinki committee (Palestinian Health Research Council) (Date: 1–6-2020\ No: PHRC\HC\702\20). The Ethics Committee of Stellenbosch University and the Helsinki committee (Palestinian Health Research Council) approved the procedure for verbal informed consent. We ensured that each participant had the right to decline participation and withdraw from the study. The privacy of the participants was protected, and their names and any identifiable information were kept confidential.

Data were collected through an online questionnaire using Google forms with a consent form. The questionnaire link was sent online on social media and papers via CBOs in Access Restricted Areas (ARAs). It was conducted from January 2021 to April 2021. The general population of the Gazan adults who fulfilled the inclusion criteria and agreed to participate in the study were included using purposive convenience and snowball sampling techniques (1000 adults) (100% responses).

Gazans of both sexes aged 18 years or older who use social media, live in restricted access areas and are willing to give informed consent were included. Those not living in Gaza or under 18 were excluded from the study.

Most individual participants included in the study provided written informed consent. However, we obtained verbal consent from [28] uneducated participants who were too old to provide written consent.

### Participants

The participants included 322 men (32.9%) and 657 women (67.1%). The mean age was 35.2 years (s.d. 11.4). Of the total, 19.5% were from the North Gaza governorate, 36.5%, 16.5%, 18%, and 9.5% from the Gaza governorate, the central area governorate, Khan Younis governorate, and Rafah governorate, respectively (see Table 1).

**Table 1:** Socio-demographic data of the study sample (*N* = 979)

Variable	N(%)
Gender	
Men	322 (32.9)
Women	657 (67.1)
Age	
18 -30 years	391 (39.9)
31–43 years	367 (37.5)
44–56 years	182 (18.6)
57–70 years	39 (4.0)
Residency place	
North	191 (19.5)
Gaza	357 (36.5)
Middle	162 (16.5)
Khan Younis	176 (18.0)
Rafah	93 (9.5)
Education level	
Uneducated	5 (0.5)
Primary	16 (1.6)
Preparatory	28 (2.9)
Secondary	192 (19.6)
University	588 (60.1)
High education	150 (15.3)
Working status	
Unemployed	447 (45.7)
Temporary work	133 (13.6)
Employer	266 (27.2)
Self-employed	25 (2.6)
Freelancing	42 (4.3)
Other	66 (6.7)
Monthly income	
Less than 1974	692 (70.7)
1974–2470	64 (6.5)
2471–2967	71 (7.3)
2968–3464	57 (5.8)
3465 and higher	95 (9.7)

### Measures

#### Demographic information

The socio-demographic variables were collected, including age, sex, marital status, area of residency, education, monthly income, and occupation*.*

#### Fear for COVID-19 scale, FCS-19 [67]

This scale was developed by Ahorsu and colleagues (2020). COVID-19 and its consequences can create a sense of fear, worry, and anxiety worldwide. The Fear of COVID-19 Scale (FCV-19S) has been validated in a sample of 717 Iranian adults. The items of the FCV-19S were constructed based on an extensive review of existing scales on fears, expert evaluations, and participant interviews. A panel reviewed the items and seven items with acceptable corrected item-total correlation (0.47 to 0.56) were retained and further confirmed by significant and strong factor loadings (0.66 to 0.74). Reliability values were acceptable; internal consistency (α = 0.82) and test–retest reliability (ICC = 0.72). FCS-19 is a reliable and robust instrument for assessing fear of COVID-19 among the general population.

#### The WHOQOL-BREF Scale [68]

The WHOQOL-BREF is a 26-item instrument consisting of four domains: physical health (7 items), psychological health (6 items), social relationships (3 items), and environmental health (8 items). It also contains QOL and general health items. Each item is scored from 1 to 5 on a Likert-type scale. The physical health domain includes mobility, daily activities, functional capacity, energy, pain, and sleep items, while the psychological domain includes self-image, negative thoughts, positive attitudes, self-esteem, mentality, learning ability, memory concentration, religion, and mental status. The social relationships domain contains personal relationships, social support, and questions related to sexual activity. Finally, the environmental health domain covers financial resources, safety, health and social services, living physical environment, opportunities to acquire new skills and knowledge, recreation, general environment (noise, air pollution, etc.), and transportation. Cronbach's alpha displayed good reliability of 0.91.

*Depression Anxiety and Stress Scale (DASS)* is a 21- items scale that assesses the emotional states of depression, anxiety and stress. The depression scale assesses dysphoria, hopelessness, devaluation of life, self-deprecation, lack of interest, anhedonia and inertia, while the anxiety scale measures autonomic arousal, skeletal muscle effects, situational anxiety, and subjective experience of anxious affect. The stress scale is sensitive to levels of chronic non-specific arousal. It assesses difficulty relaxing, nervous arousal, and being easily upset/agitated, irritable / over-reactive and impatient [69]. The scale's internal consistency was calculated using Cronbach's alpha and was 0.92.

#### Berlin Social Support Scale (BSSS)

The BSSS is a set of six scales to measure cognitive and behavioural aspects of social support [70]. The first social support dimension includes the perceived available support as the degree to which help from others is available. Secondly, the need for support is how social support is important to respondents in stressful situations. Third, support-seeking is the frequency or range of support from others that the respondent seeks. Actual support comprises the actual amount of support received from others. Provided support is a scale filled out by those who provide support to the respondent. Finally, protective-buffering support is a new construct protecting close others from bad news. This scale is filled out by the person receiving and providing support. In our scales, only support receivers were administered with the scale. Cronbach's alpha showed excellent reliability of 0.93.

### Data analysis

Statistical analysis was performed using Statistical Package for the Social Sciences (SPSS V25). Descriptive statistics were expressed by mean and Standards deviation. Frequencies and percentages are calculated and tabulated.

Hierarchical regression analysis was utilized to examine the relationship between quality of life (QoL) as the dependent variable and demographic factors (e.g., age, gender), as well as fear of COVID-19, mental health, and social support as independent variables. Significance was established at *p < 0.05 and ** p < 0.01, indicating the statistical robustness of the findings.

## Results

In total, 1000 individuals filled out the questionnaire in this study. Twenty-one questionnaires had more than 20% missing data and thus were excluded from the study. The analysis was restricted to the remaining 979 respondents. The characteristics of the study population are shown in Table 1. In Table 2, we reported the descriptive statistics of all the measures used in the study.

**Table 2:** Descriptive statistics of the study measures

	Mean	Standarddeviation	Kurtosis	Skewness
WhoBref-Physical Health	62.3	14.4	-0.08	0.02
WhoBref-Psychological Health	60.04	16.81	-0.62	0.27
WhoBref-Social relationship	62.6	20.5	-0.71	-0.18
Dass-Depression	33.14	23.6	-0.2	0.6
Dass-Anxiety	29.5	23.5	0.7	-0.1
Dass-Stress	39.9	23.6	-0.14	-0.7
FCS-19	56.7	19.3	-0.62	0.16
BSSS-Perceived emotional	76.14	21.14	-0.66	-0.56
BSSS-Perceived instruments	70.60	22.96	-0.96	-0.3
BSSS-need for support	71.77	14.8	-0.14	-0.36
BSSS-support seeking	68.91	19.51	-0.7	-0.1

*Note*: *Who-Bref* WHO Quality of life index-bref, *Dass* Depression, anxiety, stress scale, *FCS-19* Fear of COVID-19 Scale, *BSSS* Berlin Social Support Scale

Pearson's correlation coefficient was computed to assess the relationship between quality of life and the following variables: Gender, Age, Income, educational level, place of residence, type of family, Depression, Anxiety, Stress, Fear of COVID, perceived emotional support, Perceived instrumental support, Need For Support, Support Seeking.

The correlational analysis showed that QOL was positively associated with perceived emotion, instruments, and support seeking. Depression, anxiety, stress, and fear of COVID were negatively associated with high quality of life. The results also indicated that higher income and educational level were significantly associated with higher QOL. In contrast, gender was significantly associated with lower QOL (See Table 3 for details).

**Table 3:** Correlation between the study variables

	**1**	**2**	**3**	**4**	**5**	**6**	**7**	**8**	**9**	**10**	**11**	**12**	**13**	**14**	**15**
QOL	1														
Gender	-0.11**	1													
Age	0.02	-0.15**	1												
Income	0.29**	-0.08*	0.19**	1											
Educational level	0.22**	-0.16**	-0.015	0.29**	1										
Residence	-0.03	0.00	-0.03	-0.1**	-0.13**	1									
Type of family	-0.11**	0.01	-0.08**	-0.10**	-0.078*	0.02	1								
Depression	-0.52**	0.1**	-0.18**	-0.19**	-0.19**	0.01	0.00	1							
Anxiety	-0.46**	0.11**	-0.12**	-0.18***	-0.169**	-0.03	0.02	0.78**	1						
Stress	-0.42**	0.14**	-0.159**	-0.163**	-0.167**	-0.02	0.02	0.87**	0.78**	1					
Fear of COVID	-0.40**	0.051	0.048	-0.176**	-0.27**	0.12**	0.05	0.29**	0.34**	0.29**	1				
Perceived emotional	0.49**	0.017	0.04	0.11**	0.10**	-0.005	-0.04	-0.36**	-0.27**	-0.30**	-0.25**	1			
Perceived instruments	0.49**	0.007	-0.003	0.135**	0.135**	0.00	-0.06*	-0.37**	-0.26**	-0.32**	-0.22**	0.83**	1		
need for support	0.055	0.05	-0.02	-0.00	-0.01	0.01	0.05	0.03	0.01	0.082**	-0.02	0.30**	0.38**	1	
support seeking	0.24**	-0.02	0.00	0.02	0.03	-0.02	0.00	-0.142**	-0.076*	-0.08*	-0.12**	0.54**	0.55**	0.59**	1

^*^ p < 0.05. ** p < 0.01

Hierarchical regression analysis was conducted to identify the predictors of QOL. Four different models were examined to understand which predictor explains the variance. Table 4 summarizes four models, all of which were significant.

**Table 4:** Hieratical Regression with QoL as a dependent variable

	**Model1**				**Model2**				**Model3**				**Model4**			
**Variable**	**Adjusted R2**	**B**	**SEB**	**β**	**Adjusted R2**	**B**	**SEB**	**β**	**R2 adjusted**	**B**	**SEB**	**β**	**Adjusted R2**	**B**	**SEB**	**β**
	0.113				0.336				0.398				0.481			
**Gender**		-2.303	0.970	-0.073		-1.410	0.844*	-0.045		-1.473	0.805*	-0.047		-1.911	0.752*	-0.061
**Age**		-0.057	0.042**	-0.043		-0.160	0.037**	-0.120		-0.121	0.035**	-0.091		-0.103	0.033**	-0.077
**Income**		2.694	0.352*	0.248		2.054	0.307*	0.189		1.830	0.293*	0.168		1.681	0.273*	0.154
**Educational level**		2.458	0.583	0.136		1.062	0.510*	0.059		0.172	0.494*	0.010		0.091	0.460*	0.005
**Place of residence**		0.169	0.490*	0.011		-0.095	0.424*	-0.006		0.308	0.406*	0.019		0.186	0.378*	0.012
**Type of family**		-2.595	1.017	-0.078		-2.986	0.881**	-0.089		-2.685	0.840*	-0.080		-2.142	0.784**	-0.064
**Depression**						-0.218	0.034**	-0.350		-0.220	0.032**	-0.353		-0.134	0.031**	-0.215
**Anxiety**						-0.039	0.029**	-0.063		0.005	0.028**	0.008		-0.024	0.026**	-0.039
**Stress**						-0.066	0.033**	-0.106		-0.053	0.031**	-0.085		-0.043	0.029**	-0.068
**Fear of COVID-19**										-0.210	0.021**	-0.274		-0.172	0.020**	-0.225
**Perceived emotional**														0.129	0.030**	0.185
**Perceived instruments**														0.095	0.028**	0.148
**Need for support**														-0.053	0.029**	-0.054
**Support seeking**														0.025	0.025**	0.032

^*^ p < 0.05. ** p < 0.01

The first model with an Adjusted R square of 0.113 suggests that gender, Age, Income, Educational level, residence, place of residence and type of family account for 11.3% of the variance in self-rated QOL.

In the second model, we introduced depression, anxiety, and stress as dependent variables; the results indicate a noticeable improvement, where the Adjusted R-square value of 0.336 suggests that model 2 accounts for 33.6% of the variability.

In Model 3, Fear of COVID-19, the Adjusted R-value increased from 0.336 to 0.398. Such results suggest that model 3 can account for 39.8% of the variance in self-rated QOL by sample subjects. However, not all the variables in model 3 were significant.

In Table 4, we reported the specifics regarding each regression model and the associated residuals. For better illustration, it shows the coefficients of the significant variables included in the models. Nine variables in model 3 were significant, with one variable, Anxiety, which did not impact QOL.

In Model 4, Perceived emotional, perceived instruments, Need for support, Support seeking, the results indicate a significant increment, where the adjusted R-square value of 0.481 suggests that model 4 accounts for 48.1% of the variability.

When all models were examined, Fear of COVID-19 was the best predictor of QOL by explaining 22.5% of the total variance explained. Depression was the second, explaining 21.5% of the variance. Perceived emotional support was the third predictor, explaining 18.5% of the total variance. Income was the fourth predictor, explaining 15.4% of the variance. Perceived instruments was the fifth predictor by explaining 14.8% of the total variance explained.

## Discussion

This study aimed to investigate the predictors of the quality of life during the COVID-19 pandemic in the Gaza strip. We examined the association between QOL, demographic data, fear of COVID-19, psychological distress, and social support. We found that the participants reported a moderate quality of life during the COVID-19 pandemic, which was positively associated with social support and income and educational level and negatively associated with psychological distress, gender, place of residence, and family type.

Social support was positively associated with quality of life, as supported by previous studies that found that a high level of social support was associated with a high level of quality of life [71–77]. Social support might improve people's quality of life during uncertainty, including during infectious disease outbreaks [78–80]. In the case of the Gaza Strip, social networks usually support individuals living in poverty and under siege [81]. Gaza is a collectivistic society, and large families can mitigate the effect of limited individual resources but even provide socio-emotional resources that can help conserve good functioning and a subjective sense of QoL despite the deterioration of living conditions [81].

Furthermore, the study findings show that psychological distress (depression, anxiety, and stress) was negatively associated with QoL. [82–84] (H2). Examining the relationship between psychological distress and QoL during the COVID19 outbreak among health workers, Fino and colleagues [53] found that quality of life was negatively associated with depression, anxiety, and stress with minor to medium effects. Similar findings were found in other studies that targeted the general population [76, 85–87], children, and adolescents [88–92]. Regarding the Gaza context, living under siege affects the civil population's mental health and perceived QoL, mainly in conditions of isolation with scarce opportunities to fulfil basic needs and aspirations (92a; 92b). Also, the COVID-19 isolation policies imposing social distancing and 'stay at home orders contributed to deteriorating the QoL of people and increasing stress and depressive reactions. Losing social support might have contributed to diminishing QoL and exacerbating mental health conditions among Gazans.

Furthermore, lower-educated and poorer participants had lower QoL scores which are in keeping with previous research [93–95] (H4). Education is a strategic dimension of QoL in Palestinian society, reaching a literacy level of up to 98% [96]. Despite the scarcity of resources, many Palestinian families believe education is pivotal in improving life circumstances by providing work opportunities and meaningfulness. On the other hand, lower education is associated with lower life satisfaction, even during the pandemic [97]. Few studies have estimated the relationship between the level of family income and QoL, especially during crisis times. In the current study, lower-income participants reported a lower level of QoL. This result was consistent with the findings of other studies [72, 98–101] which reported that family income was the most important factor affecting people's QOL (the low income has been associated with worse QoL). In Gaza, the economic deterioration has affected most Palestinian families. During the COVID-19 outbreak, the economic isolation and crisis worsened and contributed to an ulterior decline in QoL [22].

Our study also found that gender was associated negatively with QoL (H5). This result is consistent with evidence that socio-demographic factors significantly predict QoL in general people. Seemingly, women living under challenging conditions were more affected than their male counterparts.

Finally, the hierarchical regression confirmed that COVID-19 might have been an added burden for the Palestinian population (H3). The fear of COVID-19 term added a 6.2% variance in QoL. In the final analysis, all predictors were statistically significant, with the fear of COVID-19 term recording a higher contribution of 22.5%, followed by depression term with 21.5%, perceived emotional 18.5%, income at 15.4%, and perceived instruments at 14.8% towards QoL.

Our work provides a unique picture of the Gaza population QoL during the pandemic, shedding light on the burdens of disrupted living conditions among populations affected by ongoing social suffering, conflict and military violence.

## Limitations of the study

The sample distribution was unbalanced because the data were collected through social media. For example, the percentage of women in the sample was twice that of men; the percentage of the elderly was small compared to young people, in addition to the sample's high social and educational status.

One constraint to be aware of is the utilization of adapted instruments. Although they underwent Arabic validation, none of them were specifically validated in a Palestinian context, except for the fear of COVID-19 scale. The instruments' trustworthiness may be questioned due to the lack of validation. Nevertheless, the Arabic adaptations of the measures can ensure a certain level of credibility, having been verified in related cultures.

Another limitation concerns the two different ways of data collection strategy that could have created a bias in the data analysis. However, we must consider that people with lower socioeconomic and educational backgrounds could have less confidence in dealing with technologies and find it more appropriate pencil and paper procedures. We are at risk of generalizing from the results, given these limitations. Due to the cross-sectional nature of our research design, we must consider our work exploratory and non-generalizable.

## Conclusion

The study's findings can contribute to understanding the role of fear of COVID- 19, psychological distress, and social support in the people's quality of life during the pandemic in the Gaza strip. The study displayed the vital role of the variables (fear of COVID-19, depression, perceived emotional, income, and perceived instrumental social support) towards QOL of Gazan people during the COVID-19 pandemic. The study is among the first to examine the combined psychosocial consequences of the COVID-19 pandemic and the stresses of living in the Gaza strip on quality of life. To this extent, the results may help mental health professionals understand better the pandemic's mental health consequences when working with clients who present with psychological distress. Further research can include intervention studies to improve the quality of life under conditions of uncertainty, including in war-affected areas.

The COVID pandemic has shown an urgent need for Gaza to lift the more than one-decade military block that constrains the population in the small area of Gaza. Practitioners and policymakers must consider the severe violation of human rights when designing psychosocial interventions in the COVID-19-related mental health crisis [102]. If human rights violations are not recognized, the population's mental health will remain at risk of deterioration.

## Data Availability

The datasets used and/or analyzed during the current study are available from the corresponding author upon reasonable request.

## Abbreviations

ARAs: Access Restricted Areas
BSSS: Berlin Social Support Scale
CBOs: Community-based organizations
DASS: Depression Anxiety and Stress Scale
FCS-19: Fear for COVID-19 scale
Opt: Occupied Palestinian territory
PTSD: Posttraumatic stress disorder
QoL: Quality of Life
